# Chelating Agents and the Regulation of Metal Ions

**DOI:** 10.1155/MBD.1994.87

**Published:** 1994

**Authors:** Robert A. Bulman

**Affiliations:** 1 61 Oxford Crescent, Didcot Oxfordshire OX11 7AL United Kingdom

## Abstract

Up to about the early 1980s it was perhaps still possible to summarize in a review of a moderate length the development of the medicinal applications of chelation chemistry and the exploitation of such chemistry in regulating the metal ion concentrations in the body. However, in the last few years there has a great surge in the development of chelation chemistry and its usage in medicine and related areas of life sciences research. It is no longer the case that such a review primarily concentrates upon the use of chelating agents in removing toxic metals from the body but it must now cover the use of chelating agents in the imaging procedures nuclear medicine and  magnetic resonance imaging (MRI), the use of chelating agents in unravelling the biochemistry of reactive oxidative species (ROS) and the control and measurement of intracellular calcium ions.  It is in the recent applications that there have been the greatest developments over the last ten years.


### CHELATING AGENTS AND THE REGULATION OF METAL IONS

Robert A. Bulman

61 Oxford Crescent, Didcot, OX11 7AL, Oxfordshire, England

**ABSTRACT**
Up to about the early 1980s it was perhaps still possible to summarize in a review of a moderate length the development of the medicinal applications of chelation chemistry and the exploitation of such chemistry in regulating the metal ion concentrations in the body. However, in the last few years there has a great surge in the development of chelation chemistry and its usage in medicine and related areas of life sciences research. It is no longer the case that such a review primarily concentrates upon the use of chelating agents in removing toxic metals from the body but it must now cover the use of chelating agents in the imaging procedures nuclear medicine and magnetic resonance imaging (MRI), the use of chelating agents in unravelling the biochemistry of reactive oxidative species (ROS) and the control and measurement of intracellular calcium ions. It is in the recent applications that there have been the greatest developments over the last ten years.

**INTRODUCTION: THE CONTINUING DEVELOPMENT OF CHELATION CHEMISTRY IN MEDICINE**
The essentiality of several trace metals for our good health is well recognized but in more recent years many metal compounds have also been recognized as environmental and work-place carcinogens (1, 2 and reviews therein). These observations, the use of chelated metal ions in non-invasive diagnostic medical procedures, such as nuclear medicine and MRI, the need to control the levels of toxic metal ions in the body and the search for inorganic drugs other than *cisplatin* and *auranofin* have led to extensive developments in chelation chemistry. Although inorganic drugs are always going to be minor components of a pharmacopoeia, it is possible that they might become important therapies for some significant diseases such as diabetes which in western industrial nations has an incidence of 1 in 300 (3). Although insulin replacement is the easiest method of controlling chronic diabetes the only satisfactory route of administration is by injection and this has prompted the search for orally deliverable therapies. Currently much interest has been shown in the inorganic biochemistry of vanadium as in some forms it is an orally active insulin mimetic (4). Although vanadate is active, its uptake from the gastrointestinal tract is low but vanadyl bis-maltolato is orally active and is a powerful insulin mimetic (5). The recently reported metabolic problems ascribed to vanadium in Thais dwelling in regions with vanadium-rich soils (6) should be studied in depth before the launch of vanadium- containing pharmaceuticals.

It is clear from studying the literature on inorganic biochemistry in its widest forms, from the speciation of metal ions to investigations of the clearance of toxic metal ions from the body, that many novel approaches have been used to modify the behaviour of the biological response to metal ions. Although low molecular weight chelating agents have been the major mechanism for modifying biological response to metal ions, other procedures which exploit metalloproteins are almost certainly going to become feasible as genetic engineering develops. Already of note has been the production of an antibody which bears a functioning metallothionein which can be loaded with cationic $^{99m}Tc$ and then targeted to cells (7). The recent considerations about metallothionein and cancer (8) might lead to further investigations of such genetic engineering.

The reasons for giving serious consideration to the contribution of inorganic elements to mammalian biochemistry continue to grow and it is clearly unwise to assume there is a full understanding of mammalian biochemistry because of the use metabolic pathways as vanity boards in laboratories and offices. The continuing development of analytical instrumentation is likely to lead to the identification of more essential trace elements or the concentration of elements in diseased tissue. For instance, by using particle induced X-ray emission spectroscopy strontium was identified in the granulocytes of patients with rheumatoid arthritis and shown to fall from 5.1 µg/g dry weight to 1.7 µg/g over a three month course of treatment with anti-inflammatory drugs (9). The extent to which chelating agents have continued to be the subject of study in biochemistry and medicine can be judged from Table 1 which was compiled from an on-line search of *Medline* using the search terms *chelat** and the names of various chelating agents. Unfortunately because of the way the files on the *Medline* database are currently constructed it is impossible to summarize the data for a sequence of, say five years, by other than an much more expensive search. By using some additional terms it has also been to obtain some idea of the number of publications in which D-penicillamine was likely to have been discussed primarily in the treatment of rheumatoid arthritis. From the number of hits for 1985-89 and from 1990 to July 1993 it is clear that research applications of chelating agents in medicine and the relevant life sciences shows no sign of declining but is actually increasing. By the mid-1960s the initial use of chelating agents in medicine as almost a panacea had practically stopped, so it might be presumed that the hits for the years from 1966 onwards primarily reflect accounts of their use for chelation of metal ions in medicine and the relevant life sciences. No doubt many of these uses in life sciences reflect the development of inorganic biochemistry.

Table 1 Summation of hits from an on-line search of *Medline* for the various chelating agents listed below

| Years | NUMBER OF HITS FOR THE SEARCH TERMS BELOW | | | | | | | | | | |
|---|---|---|---|---|---|---|---|---|---|---|---|
| | Chelating agent | EDTA* | DTPA | DFOA | DMSA | DMPS | BAL | DDC | PEN | PEN# Chelat | PEN# Rheuma |
| 66-84 | 21950 | 14429 | 1388 | 954 | 247 | 40 | 100 | 46 | 1279 | 1162 | 467 |
| 85-89 | 7109 | 4070 | 1558 | 917 | 235 | 49 | 28 | 47 | 498 | 399 | 192 |
| 90-93 | 7639 | 3529 | 1985 | 915 | 314 | 49 | 31 | 48 | 338 | 221 | 104 |

* Respectively ethylenediaminetetraacetic acid, diethylenetriaminepentaacetic acid, desferrioxamine, 2,3-di-mercaptosuccinic acid, 2,3-dimercaptopropane sulphonic acid, 2,3-dimercaptopropanol, diethylenedithio-carbamic acid and D-penicillamine. Note *BAL* was not used as the search term but instead the search term was *dimercaptopropanol*; # Combined respective search terms D-PEN + *chelat* and D-PEN + *rheuma*

In this account the development of chelation chemistry is summarized by highlighting its exploitation in the following uses:

(a) As counter measures to the uptake of toxic metals, uses reviewed over many years and summarized elsewhere in 1987 (10) and in 1991 (11,12).

(b) In non-invasive diagnostic medicine as vehicles for the transport of radionuclides such as $^{99m}Tc$, $^{67}Ga$, $^{111}In$ used in nuclear medicine and more recently paramagnetic species, primarily $Gd^{3+}$, in MRI;

(c) As targeting agents in radiotherapy, particularly when bound to macromolecules such as monoclonal antibodies;

(d) As anti-microbials, anti-malarials, anti-virals, anti-neoplastic agents and anti-parasitic agents;

(e) In investigations of free radical chemistry.

### OCCURRENCE OF CHELATING AGENTS

It is well recognized that many low molecular weight chelating agents of considerable diversity occur in nature and practically all of the more common chelating moieties, other than dithiocarbamate and vicinal dithiols, have been observed in nature, particularly as extracellular products of the protista. Even the N-$CH_2COOH$ moiety of the polyaminopolycarboxylic acids (PAPCs) and 2,2'-bipyridyl are to be found in nature in pyridine-2,6-dicarboxylic acid and bipyridyl-containing caerlomycin (13). In a recent extensive review, well worth detailed study, Michael and Pattenden have taken a critical look at potential metal ion chelation by marine metabolites (14). In man regulation of the concentration of metal ions is achieved primarily by uptake by macromolecules such as transferrin, ferritin, metallothionein, caeruplasmin and, rather weakly, by serum albumin. Regulation of metal ions by low molecular weight chemicals appears to be primarily by citrate and amino acids. The extent to which an interaction of adrenaline, the possessor of the o-catechol moiety, with copper(II) and iron(III) has any significance in our wellbeing, or perhaps lack of it, is still to be demonstrated as catecholamines are prone to autooxidation in the presence of transition metal ions. The occurrence of 3,4-dihyroxyphenylalanine in the primary sequence of some proteins has been noted (15) but the participation of this moiety in metal-binding has yet to be determined. The occurrence of vanilmandelic acid in neuroblastoma has been noted (16) as has the occurrence of a siderophore-growth factor in virally transformed cells (17). A role for higher aliphatic 2,4-diketones, a ubiquitous lipid family with chelating properties, is still being sought, although it is recognized that they exhibit anti-allergic and antihistamine properties (18).

### METAL IONS IN CELLS

As Sorenson (19) has indicated, complexes and chelates of metal ions are the biologically relevant forms of the metalloelements as they produce identifiable pharmacological effects. The concentrations in plasma of essential transition trace elements, as free ions, are low and have had to been estimated by computer simulation studies (20). Of the major potentially chelatable cations in plasma the concentration of $Mg^{2+}$ and $Ca^{2+}$ is several orders of magnitude higher (20) than the next most abundant species listed in Table 2. Also summarized in Table 2 is the amount of the element in a 70 kg standard man and the number of atoms per cell (21). It is presumed that the concentrations of ionically bonded transition metal ions in cells are much lower than the concentrations in plasma as cells have much higher concentrations of covalent and coordinate-covalent bonding ligands.

Table 2 Amounts of the major chelatable elements in plasma, in cells and in standard 70 kg man

| | Mg | Ca | Mn | Fe | Cu | Zn |
|---|---|---|---|---|---|---|
| Molarity in plasma | 5.2 E-4 | E-3 | E-12 | E-23 | E-18 | E-9 |
| Grams per 70kg | 35 | 1050 | 0.02 | 4.2 | 0.11 | 2.33 |
| No of atoms per cell | 8.7 E9 | 1.6 E11 | 2.2 E12 | 4.5 E8 | 1 E7 | 2.2 E8 |

### METAL ION REGULATION BY DRUGS NOT SPECIFICALLY DESIGNED AS CHELATING AGENTS

Perturbations of metal ions during medication are recognized and in some cases might be the unwanted side-effects of the drugs. In general organic drugs, other than chelating agents, are not designed to have appreciable metal-binding properties and little attention has been paid to the perturbation of metal ion concentration through normal medical treatment (22). Although changes in trace element concentration can be the intended result of therapy, they are more often the result of side-effects which are unintentional sequellae of the therapy. No doubt there are many unrecognized alterations in trace element concentrations during treatment and presumably these concentrations return to normal after the end of the treatment. Alterations in trace element concentrations are only likely to be noted in cases where the therapy is very long term or if the side-effect is so drastic that it manifests itself early in the treatment. All organic compounds containing heteroatoms that have donatable lone pairs of electrons are potentially capable of interacting with metal ions. By and large the rigorous testing procedures used in drug development should not lead to the marketing of drugs with such unwanted side effects as major or long term alterations in the concentrations of trace element. By determining the formation constant of a complex formed between a potential drug and a series of metal ions and also knowing the formation constants for the complexes formed between metal ions and biochemical ligands, it is possible to make predictions of the extent to which the chemical might alter the normal distribution of metal ions in fluids such as plasma. These procedures became available in the late 1970s (20) when computer programs were developed which permitted high speed calculations to be run so that the distribution of metal ions between several compartments could be determined. These computer simulation models have shown EDTA in plasma exists primarily as the calcium chelate with the exception of 15% present as the zinc chelate (23). In contrast, DTPA exists primarily in plasma as the zinc chelate.

Only a few cases of the interaction drugs with metal ions are given here as this lecture is primarily a summary of the biomedical applications of chelating agents. Acquirement of anti-inflammatory properties by anthranilic acid and 3,5-diisopropylsalicylic acid arises only on complexation of copper(II) while other anti-inflammatories such as aspirin and D-penicillamine are more active as their copper(II) complexes (24). The chelation of essential trace elements by salicylates might well become significant developments worth using in radiotherapy. Currently studies by Sorenson are indicating that complexes of first row transition metal ions with anti-inflammatories, such as 3,5-di-isopropylsalicylate, can increase dramatically the survival rate of gamma-irradiated mice (25).

Although metal ion-binding functions are not normally significant moieties in drugs, they have been incorporated into the structure of the non-steroidal anti-inflammatory drugs which generally exert their effect by binding copper(II). Isoxicam (Fig. 1a), now withdrawn because of its purported side-effects, possesses the ß-diketone metal-binding moiety as well as the sulphoxide ligand (26). Treatment of epilepsy with phenytoin (Fig. 1b) leads to alterations in copper and zinc metabolism (27). Interestingly, a few years before this report, Weismann et al (28) had reported a greater oral uptake and greater daily loss of zinc by rats fed phenytoin at levels far in excess of therapeutic levels.

The anti-ulcerogenic drug cimetidine possesses several ligands and it is been shown that its binding of $Cu^{2+}$ greatly enhances its binding to receptor sites on the rat brain (29). The possession of such a wide range of ligands no doubt contribute to some of the recently reported biochemical properties of cimetidine (30). $Cu^{2+}$- and $Ni^{2+}$-binding properties have been reported for famotidine, a structurally similar anti-ulcerogenic drug (31).

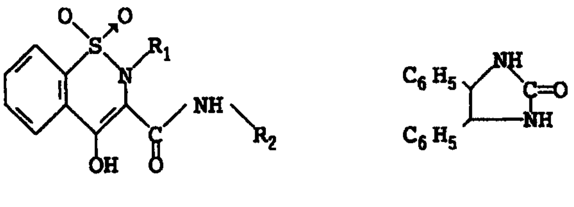

Fig. 1ab Structure of isoxicam and phenytoin

Perturbation of the levels of essential metal ions might not necessarily arise directly from complexation by drugs but might arise from elevation of metal ion-binding proteins such as metallothionein. Several agents, as diverse as whole body X-ray exposure (32) to induction by nonstearoidal antiinflammatory drugs (33) and the $Ca^{2+}$ ionophore calcimycin (34) elevate metallothioneins levels. The induction in a yeast of cadystins, metallothionein-like peptides, by DDC (35) might point to a similar induction of metallothionein in animals.

### THE DEVELOPMENT OF CHELATING AGENTS AND THE MOBILIZATION OF METAL IONS

The use of chelating agents to counter the toxic action of metal ions began about fifty years in England when 2,3-dimercaptopropanol was developed to counter arsenic-containing nerve gases. In the twenty years or so that followed various other chelating agents and covering the full spectrum from the hard bases, primarily EDTA and its congeners (Fig. 2), through the intermediate hardness of polyamines, such as triethylenetriamine, to the soft bases (Fig. 3) were screened for ability to mobilize a variety of toxic metal ions from the body. As inorganic biochemistry developed from the mid-1970s onwards there has been a considerable expansion in the synthetic chemistry of chelating agents and the forms in which they are used (Fig. 4). To enhance the uptake of chelating agents into soft tissues, in which toxic metal ions have concentrated, chelating agents have been used in forms such as: (i) lipophilic species, (ii) phagocytizable macromolecular chelating agents (MCAs) and (iii) hydrophilic species entrapped in liposomes or even re-sealed erythrocytes. The extensive literature covering these uses has been reviewed (10).

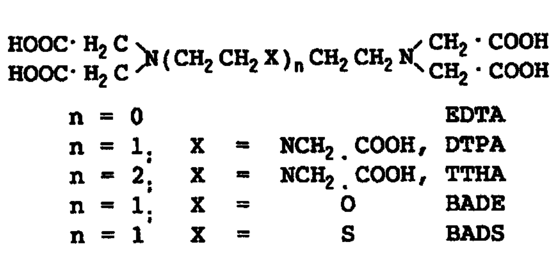

Fig. 2 Structure of EDTA and congeners

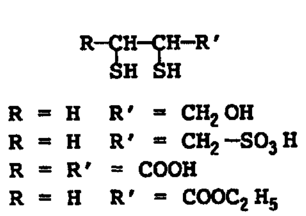

Fig. 3 2,3-Dimercaptans

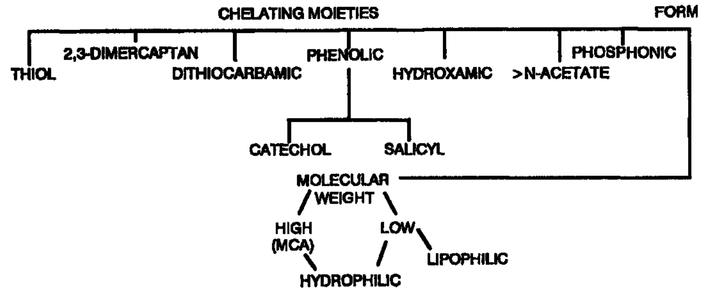

Fig. 4 The moieties of chelating agents and the forms in which chelating agents might be used

Some therapeutic and potentially therapeutic applications of MCAs in combating toxic metal ions have been briefly summarized (10). The accidental poisoning of small children who had ingested ferrous sulphate tablets has led to the preparation and evaluation of hydroxamate-and catechol-containing MCAs to block absorption of iron. By evaluation in mice it was established that MCAs of around $10^6$ Daltons formed from methacroyl moieties and bearing hydroxamate side-chains had low toxicity and were effective. An effective blocking of enterohepatic recirculation of methylmercury(II) in rural- dwelling Iraqis, who consumed grain which had been treated with a mercury-based fungicide, was achieved by using a thiol-containing macromolecule.

Chelation-based procedures for removing toxic metal ions from bone, and also directing the uptake of gamma-ray emitting radionuclides to bone, have also been sought. Whereas procedures for selective direction of some chelating agents to soft tissues have been relatively successful, and are the subject of further discussion below, the success in directing chelating agents to bone is perhaps not quite so evident. As a fuller summary of the interaction of chelating agents, and chelated radionuclides, with bone is given elsewhere (36) only a brief outline is given here. Uptake of N-acetate-type chelating agents by bone appears to be low, 0.2% uptake, and is restricted to some iminodiacetates which form calcium chelates with stability constants of log K 5. Presumably the uptake of xylenol orange and calcein blue by bone is determined by the N,N-iminodiacetate moieties they contain.

The affinity of tetracycline for calcified tissues presumably arises from keto-enol group generated by the 11,12-diketone group (37). A highly effective uptake into bone of gamma-ray emitting radionuclides, such as $^{99m}Tc$ , by using bis-phosphonates has become the basis of extensively exploited procedures for the detection of excessive bone growth due to tumours (38) and Paget's disease (39). More recently some N-substituted phosphonates derived from dimethylamino-dicyclopentadiene and dimethylaminonorborane have been evaluated and might also become important vehicles for targeting radionuclides to bone (40).

To achieve a significant manageability of the use of chelation agents in decorporation therapy, their usage has been summarized in Table 3. With the exception of HPO, the hydroxypyridones (Fig. 5), these chelating agents are listed in the pharmacopoeia of many nations. The extent to which toxic metal ions have been removed from the human body and thus been life-saving is not always easy to assess. In spite of the reservations in the USA of the use of prophylactic chelation (41-43), from laboratory studies on animals and the many cases of clinical usage, the evidence is substantial that chelation therapy to combat the intake of toxic metal ions by humans is clearly beneficial. Perhaps one of the most demonstrative studies of the benefits of chelation therapy is the marked reduction of the incidence of bone sarcomas in mice, the recipients of ZnDTPA chelation therapy to counter the radiobiological consequences of intravenously injected $^{239}Pu(IV)$ citrate (44). In humans the benefits of DFOA therapy in relieving the painful symptoms of aluminium uptake into bone have been clearly demonstrated (45). DFOA has also clearly been of value for combating aluminium-induced dialysis encephalopathy (46,47). Recently it has been reported that intramuscular injections of DFOA halted the decline in daily the living skills of patients with Alzheimer's disease (48). However, the role of aluminium in the aetiology of this disease continues to be controversial (49,50).

In the remainder of this section on the mobilization of toxic metal ions discussion will concentrate primarily on the mobilization of transuranic elements, iron and cadmium, those areas where there have been the greatest developments in chelation chemistry. The similarities in the hard acid characteristics of Pu(IV) and Fe(III) led to the evaluation of DFOA, and later other naturally occurring $Fe^{3+}$- selective chelating agents (siderophores), for ability to mobilize Pu(IV) from experimental animals. Over about the last fifteen years numerous siderophores, encompassing catechol and hydroxamic acid moieties, have been synthesized and screened for ability to mobilize cations of Pu and Am from experimental animals. Some of the key developments in the evaluation of this extensive synthetic programme, directed by Raymond at the University of California, have been briefly summarized in a recent publication by Volf et al (51), an account of early chelation therapy in the rat to counter uptakes of $^{238}Pu$ and $^{241}Am$.

Table 3 Application of chelating agents to mobilize toxic metal ions

| | CHELATING AGENTS | | | | | | | | | | |
|---|---|---|---|---|---|---|---|---|---|---|---|
| Element | BAL | PEN | AcPEN* | DMPS | DMSA | DDC | TRIEN | DTPA | EDTA | DFOA | HPO |
| As(III) | + | | | | | | | | | | |
| Au(I) | + | | | | | | | | | | |
| Hg(II) | + | | + | + | + | | | | | | |
| $CH_3$-Hg(III) | + | | | + | + | | | | | | |
| Cd(II) | | | | | | + | | | | | |
| Cu(II) | | + | | | | | + | | | | |
| Pb(II) | | + | | | | | | | + | | |
| Fe(III) | | | | | | | | + | | + | + |
| Ni(II) | | | | | | + | | | | | |
| $^{239}$Pu(IV) | | | | | | | | + | | | |
| $^{241}$Am(III) | | | | | | | | + | | | |
| Al(III) | | | | | | | | | | | |

*N-acetylpenicillamine

The success of Raymond's highly innovative work can be judged by the reduction, by continuously infused 3,4,3-LIHOPO (Fig. 6), of $^{241}$Am and $^{238}$Pu in bones to <5% and 10% of the respective levels in controls. The contents of the livers were reduced to <2% of the controls.

Raymond's contribution to chelation therapy has been marked by the syntheses of ligands of a variety of metal ions and of recent note is the synthesis of the lead(II)-sequestering bis(hydroxypyridinethione) ligand (Fig. 7) (52).

Almost parallelling Raymond's extensive syntheses of siderophore-like chelating agents has been the syntheses of chelating agents to counter the accumulation of iron in patients hypertransfused to counter thalassaemia major. One of the prime motivators of the research has been the great costs of DFOA- based chelation therapy. A detailed account of such syntheses, particularly of orally administered chelators, is unwarranted here as adequate accounts are in existence and particularly from the clinician's viewpoint (53).

The syntheses of lipophilic bidentates chelating agents such as the 3-hydroxy-pyridin-2-ones, 1-hydroxy- pyridin-2-ones and 3-hydroxy-pyridin-4-ones (11, 54) have been clearly major developments in not only countering iron overload but also in expanding the use of ferric-selective chelators in some other areas of life science research which are discussed later. Although these HPOs have already entered some early clinical evaluations, their true value as orally administrable chelating agents will require several years of clinical evaluation.

In a radical departure from the siderophore concept, Hegetscheiler and his colleagues have synthesized 1,3,5-triamino-1,3,5-trideoxy-cis-inositol (55), and the N,N-dimethyl homologue (56), and evaluated their chelation of iron(III), aluminium(III) and copper(II). This continued development of chelating agents for use in intercepting iron is clearly necessary as it may aid us in furthering our

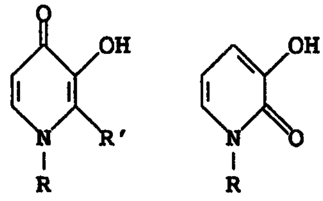

Fig. 5 Hydroxypyridones

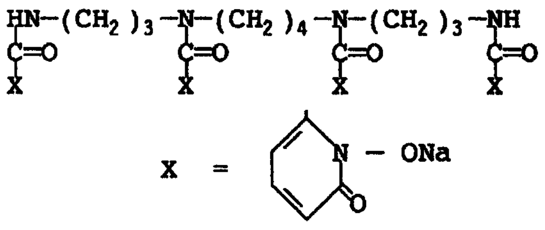

Fig. 6 3,4,3-LIHOPO

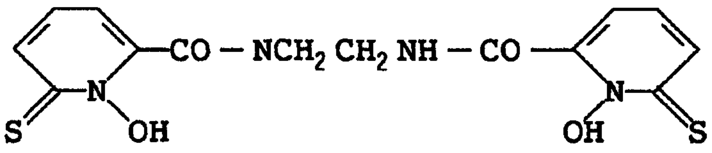

Fig. 7 [1,2-ethyanediylbis(iminocarbonyl)]bis(1-hydroxy-2(1*H*)-pyridine-2-thione knowledge of the inorganic biochemistry of iron, a need suggested by the speculations of Stevens and Kalkwarf (57). In their review they have speculated that western diets might lead to an increased iron content of cells and tissues and thus may increase the risk of radiation-induced cancer.

Jones (12) has summarized his extensive investigations of the structure-activity relationships of a series of dithiocarbamic acids prepared to counter incorporated cadmium (Table 4 and Fig. 8). As can be seen from Table 4, he has succeeded in obtaining significant reductions in the cadmium burden of kidneys and liver by fine-tuning the amphilicity of the DDC derivatives.

As the chemistry of chelating agents has developed many accounts have detailed procedures for synthesizing an ever extending array of the more common chelating agents. Foremost amongst these studies have been accounts of the preparation of new PAPCs. Early attempts at modification were no doubt hindered by the erroneous assumption that EDTA and its congeners had a limited chemistry. A variety of derivatives of EDTA and DTPA have now been prepared by formation of anhydrides and also by using reagents developed for activating carboxylic acids. The variety of PAPCs range from those bearing substituents, such as lipophilic moieties and amino acids, on the carboxylic groups (Fig. 9) to PAPCs, generally described as bifunctional chelating agents, bearing substituents on the alkyl moieties. In many cases the structural modifications of EDTA and DTPA, or in many cases the total synthesis of new forms of these chelating agents, have been applied in what has become known as *bioconjugate chemistry*, a term used to describe the coupling of small molecules to macromolecules such as polysaccharides (58,59) and proteins, primarily monoclonal antibodies (MoAbs), for which there are now numerous references (60- 64 and references therein). In the last few years the developments in synthetic organic chemistry of the EDTA- and DTPA-like molecules has become extensive and are briefly summarized in Table 5. There have also been similar developments in the chemistry of N-substituted 1,4,7,10-tetrazacycloalkanes (Table 6). The principal impetus in the development of these chelate-bearing MoAbs has been in nuclear medicine

Table 4 Clearance of cadmium from mice by DDC derivatives of varying amphilicity and varying molecular weight

| DDC | R | Z | X | n | Doseage mmol/kg | Kidney# Cd | Liver Cd |
|---|---|---|---|---|---|---|---|
| DDC-1 | Me | - | CHOH | 3 | 4.4 [9]* | 0.46 | 0.97 |
| DDC-2 | - | $C_6H_5$ | CHOH | 3 | 0.4 [7] | 0.99 | 0.96 |
| DDC-3 | - | 4-Me-O-$C_6H_5$ | CHOH | 3 | 1 [5] | 0.19 | 0.39 |
| DDC-4 | - | 4-Me-O-$C_6H_5$ | CHOH | 5 | 1[2] | 0.59 | 0.23 |
| DDC-5 | - | $C_6H_5$ | -$CH_2$CH.CHOH.$CH_2$OH<br>O.Gal§ | 1 | 0.4[5] | 0.39 | 0.38 |
| DDC-6 | Me | $C_6H_5$ | -$CH_2$CH.CHOH.$CH_2$OH<br>O.Gal | 1 | 0.4[5] | 0.39 | 0.20 |

### No therapy, control animals, so taken as 1.0; * frequency of injection; § ß-D-galactopyranosyl

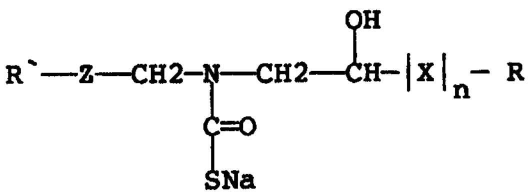

Fig. 8 Basic structure of diethyldithiocarbamic acids where there has been the quest for improved imaging of tissues and also for highly directed radiotherapies (65-67).

Further improvements in the syntheses of metal chelate-bearing proteins have continued. Of note is a recently reported procedure for incorporation of chelating moieties into rationally selected sites of proteins (68). Ebright et al (68) have achieved a high-efficiency incorporation of an EDTA-metal complex into a protein. The key to this procedure was to use site-directed mutagenesis to produce a unique solvent-accessible cysteine residue which can be derivatized by reaction with N-(iodoacetyl)-p- phenylenediamine-EDTA:metal, a novel haloacetyl derivative of EDTA:metal.

A high specificity of antibodies for In(III)EDTA-bearing proteins has been noted (69). On replacement of $In^{3+}$ by $Sc^{3+}$ or $Ga^{3+}$ on the EDTA-bearing antigen, the antibody binding was reduced by more than three orders of magnitude. This reduced interaction of the antibody indicates that proteins bearing $Sc^{3+}$ or $Ga^{3+}$ instead of $In^{3+}$ might have significantly different conformations which have been brought about by different ligands being donated to $Sc^{3+}$ or $Ga^{3+}$. Other important, and in some cases earlier (10), developments in the chemistry of the PAPCs have involved producing them in various lipophilic forms and also as nucleic acid-intercalating derivatives which could be used to fragment nucleic acids into rationally-determined nucleotide sequences.

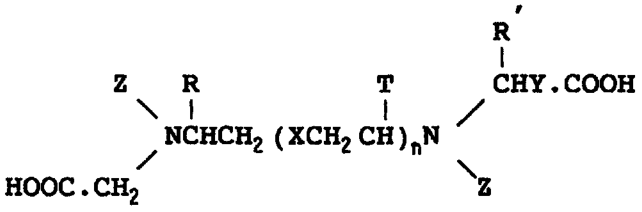

Fig. 9 Structural variations of some recently synthesized PAPCs

Table 5 Structures of some PAPCs containing either EDTA or DTPA as the chelating group

| | Z | R | n | Y | R' | T | X | REF |
|---|---|---|---|---|---|---|---|---|
| EDTA | $CH_2COOH$ | H | 0 | H | - | H | - | |
| EDTA-1 | $CH_2COOH$ | *p*-NH.$C_6H_5$ | 0 | H | - | H | - | (70) |
| EDTA-2 | $CH_2COOH$ | H | 0 | - | *p*SCN.Bz- | H | - | (71) |
| EDTA-3 | $CH_2COOH$ | -$(CH_2)_2$.COOH | 0 | H | - | H | - | (72) |
| EDTA-4 | $CH_2COOH$ | Tosyl.NH-$(CH_2)_2$ | 0 | H | - | H | - | (72) |
| EDTA-5 | $CH_2COOH$ | $(CH_3)_2$CH.CO- | 0 | H | - | H | - | (73) |
| EDTA-6 | $CH_2$CO.NH<br>HOOC.CH$CH_2$.SH | H | 0 | H | - | H | - | (74) |
| EDTA-7 | $CH_2$CO.NH<br>HOOC.CH$(CH_2)_2$.$SCH_3$ | H | 0 | H | - | H | - | (75) |
| EDTA-8 | $CH_2$CO<br>O$CH_2$.CH(SH).$CH_2$SH | H | 0 | H | - | H | - | (76) |
| DTPA | $CH_2COOH$ | H | 1 | H | - | H | $NCH_2COOH$ | |
| DTPA-1 | $CH_2COOH$ | H | 1 | $CH_2$ | - | - | $NCH_2COOH$ | (77) |
| DTPA-2 | $CH_2COOH$ | -$(CH_2)_4NH_2$ | 1 | H | - | - | $NCH_2COOH$ | (78) |
| DTPA-3 | $CH_2$CO.NH.Pr | H | 1 | H | H | H | $NCH_2COOH$ | (79) |
| DTPA-4 | $CH_2$CO.NH.Et | H | 1 | H | H | H | $NCH_2COOH$ | (79) |
| DTPA-5 | $CH_2$-C-N-CH(Me)<br>O OH | H | 1 | H | - | H | $NCH_2COOH$ | (51) |
| DTPA-6 | $CH_2COOH$ | *p*$NH_2$.Bz- | 1 | H | - | Me | $NCH_2COOH$ | (80) |
| DTPA-7 | $CH_2COOH$ | *p*SCN.Bz- | 1 | H | - | Me | $NCH_2COOH$ | (81) |

### MONITORING THE INTRACELLULAR INORGANIC BIOCHEMISTRY OF $Ca^{2+}$ BY USING CHELATING AGENTS

Calcium ions are now known to be important regulators of intracellular metabolism. Increases in the intracellular concentration of $Ca^{2+}$ increases the activities of many enzymes and also regulates various specialized cellular processes such as muscle contraction, phagocytosis and the secretion of hormones such as insulin and histamine. The unravelling of the role of intracellular calcium in processes such as phagocytosis and hormone secretion has been greatly facilitated by using two major categories of chelating agents. In the first category are the polyether carboxylic antibiotics which started to have a significant input into biochemical research at the cellular level about twenty five years ago. These naturally occurring $Ca^{2+}$-transporting ionophores, calcimycin and lasalocid, formerly A23187 and X537A, are lipophilic and thus mediate the uptake of $Ca^{2+}$ across cell membranes by an electroneutral mechanism, in other words there is no energy-dependent transport of $Ca^{2+}$ across cell membranes. In the second category are several PAPCs which are taken up by cells as the easily hydrolyzable acetoxymethyl esters. This type of membrane-permeable PAPC (Fig. 11) was originally synthesized by Tsien (85) with the aim of producing a $Ca^{2+}$-selective fluorescing chelating agent for quantitation of intracellular $Ca^{2+}$. The applications of these various chemicals have become numerous and much interesting data on the inorganic biochemistry of calcium ions has been generated. By such procedures it has been possible to show young erythrocytes contain 62 nM $Ca^{2+}$ whereas much older cells contained 221 nM $Ca^{2+}$ (86).

In more recent developments the $Ca^{2+}$-selective PAPCs have been extended by preparation of forms which can be photochemically activated inside cells and so produce controlled fast decrements in intracellular concentrations of free $Ca^{2+}$ (87). By using fluorescence microscopy it is possible to measure cytosolic $Ca^{2+}$ in individual cells loaded with these chelating agents (88). By attaching a micro televison camera to a fluorescence microscope, Mohri and Hamaguchi (89) captured as series of coloured images the propagation of transient $Ca^{2+}$ increase in sea urchin upon fertilization. Inevitably there have been reports of the need for caution in ascribing some results to the actions of these intracellular PAPCs (90). Modifications of the structure of chelating agents has not been primarily restricted to PAPCs. By derivatizing DFOA to N-stearoyl-DFOA the chelating agent became sufficiently lipophilic that it underwent biliary clearance (91). In more recent years DFOA has been coupled to biocompatible polymers to prolong circulation in blood (92). Several groups have synthesized mono- and diesters of dimercaptosuccinic acid and evaluated the derivatives for removal of lead and cadmium (93). A recent review (94) has summarized the uses of DMSA and DMPS in the People's Republic of China where DMSA has also been used to treat intoxications by pesticides and mushrooms.

### NUCLEAR MEDICINE

Nuclear medicine has been one of the great boons of the nuclear age. The principal radionuclide used in imaging radiopharmaceuticals is $^{99m}Tc$, $t_{½}$ 6.02 h, the decay product of $^{99}Mo$, $t_{½}$ 66 h, which is retained as molybdate on an alumina column. After elution as the pertechnate ion, $TcO_4^-$, in sterile saline, various reducing agents are used in the presence of a chelating agent, although occasionally the ligand is not a chelating agent, to yield radiopharmaceuticals containing Tc(V), Tc(VI), Tc(III) or Tc(I). By selection of suitable ligands it is possible direct the radionuclide to various organs or diseased tissue and, also, ensure a rapid high uptake of the radionuclide into the target tissue into contrast to a continuing circulation in blood. The contribution of slight structural variations of a series of acetanilidoiminodiacetates upon the distribution of $^{99m}Tc$ in mice has been summarized (95). Also of note is an extensive study of the species-dependent distribution of $^{99m}Tc$ in various oxidation states when complexed by 1,2-bisdimethylphosphinoethane (96). Further discussion of the use of chelating agents in nuclear medicine appears elsewhere (10). A detailed account of the coordination chemistry of radiopharmaceuticals has recently been issued (97). Although $^{99m}Tc$-containing radiopharmaceuticals are the major chemicals used in nuclear imaging of the body other chelated radionuclides (principally $^{67}Ga(III)$ and $^{111}In(III)$) are also used. The recent development of other parent/daughter generators, $^{188}W/^{188}Re$, $^{62}Zn/^{62}Cu$, $^{68}Ge/^{68}Ga$, $^{90}Sr/^{90}Y$ and $^{115}Cd/^{115m}In$, extends

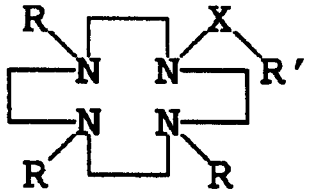

Fig. 10 1,4,17,10-Tetraazacyclododecane

Table 6 Substituted 1,4,17,10-Tetraazacyclododecane PAPC analogues

| | R | X | R' | Ref |
|---|---|---|---|---|
| DOTA* | $CH_2COOH$ | - | $CH_2COOH$ | - |
| DOTA-1 | $CH_2COOH$ | $CH_2$ | Me.CH(OH) | (82) |
| DOTA-2 | $CH_2COOH$ | $CH_2$ | $CH_2$(OH).CH(OH) | (82) |
| DOTA-3 | $CH_2COOH$ | $CH_2$ | $CONH_2Me$ | (82) |
| DOTA-4 | $CH_2COOH$ | $CH_2$ | $CONH_2CH_2CH_2$.O H | (82) |
| DOTA-5 | $CH_2COOH$ | $CH_2$ | $CONHCH_2CH_2$.Me | (83) |
| DOTA-6 | $CH_2$Po(Me)OH | - | $CH_2$PO(Me)OH | (84) |
| DOTA-7 | $CH_2$P(Me)OH | $CH_2$ | CO.NHMe | (84) |
| DOTA-8 | $CH_2$PO(Bu)OH | $CH_2$ | CO.NHMe | (84) |
| DOTA-9 | $CH_2PO(C_6H_5)OH$ | - | $CO.N(CH_3)_2$ | (84) |

* 1,4,7,10-tetrakis(carboxymethyl)-1,4,7,10-tetraazacyclododecane the diversity of the ligands which might be used to form imaging radiopharmaceuticals and radiotherapies. In the last few years nuclear medicine has expanded further by using more selective procedures to target radionuclides to tissues by using chelating agents chemically immobilized on monoclonal antibodies. Although some of these MCAs have been used to improve targeting of nuclear imaging, they have mainly been developed for radiotherapy. Torchilin and Kilbanov (98) have recently reviewed the applications of antibody-linked chelating polymers in radiotherapy and diagnosis.

### MAGNETIC RESONANCE IMAGING

As is obvious from the account of the syntheses of many new PAPCs, the development of MRI has led to many publications of the screening of chelating for use in MRI. Estimates have put the contrast agent pharmaceuticals sales at around $2 billion (99). Several types of paramagnetic metal ion-chelate complexes (magnetopharmaceuticals) have been examined as contrast-enhancing agents in MRI. By chelating paramagnetic metal ions it is possible to reduce their toxicity and also target the paramagnetic ion to various organs in the body. By chelation of Gd(III) by ligands such as DTPA it is possible to raise its LD50 in mice from 0.38 mmol/kg (84) to more than 5 mmol/kg (99). Currently chelated Gd(III) is the principal paramagnetic species used to form magnetopharmaceuticals and several Gd(III)- containing magnetopharmaceuticals, such as the DTPA and DTPA-diglucamide chelates, have been cleared for use clinical trials (100, 101). Chelated Mn(II) has also been used in humans (102). These hydrophilic agents are rapidly excreted by glomerular filtration through the kidneys into urine with a biological half-life of 90 minutes. These hydrophilic chelates enhance brain tumours because they are fed by capillaries which lack a blood-brain barrier, the tissue which blocks passive diffusion of hydrophilic substances into neural tissue.

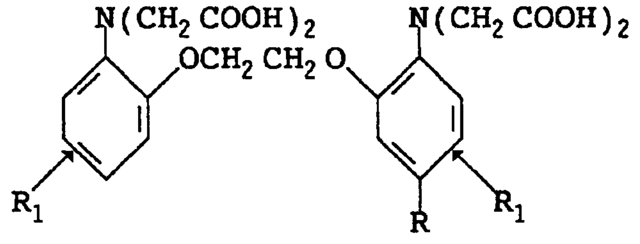

Fig. 11 Basic structure of $Ca^{2+}$-binding chelates which fluoresce

Chelated Gd(III) has also been used in various lipophilic DTPA forms which are cleared to the liver and biliary duct (103-105) and as a variety of DTPA-bearing MCAs (106-108). By using antisera raised to various forms of DTPA chelates of Gd(III) evidence has been obtained which indicates that Gd- DTPA is possibly bound by serum proteins (106). Four cases of anaphylactic response to Gd-DTPA substantiate (109) this observation. As DTPA-based ligands are not totally kinetically inert dissociation of the chelates can result in accumulation of Gd(III) in bone and liver. In the quest more stable forms of Gd(III)-containing magnetopharmaceuticals, Parker's group at the University of Durham have demonstrated kinetic stability of various phosphinic acid analogues of DOTA (84). In contrast to these DOTA derivatives, Sessler et al (110) have achieved kinetically inert complexes of Gd(III) by using *texaphyrins*, porphyrin-type macrocycles.

The preparation of such kinetically inert chelates will greatly extend the use nuclear magnetic resonance procedures in medicine. Just as lanthanide shift reagents were of great benefit in evaluating the structures of organic chemicals it can be expected that their introduction into diagnostic medicine will be equally beneficial. Already the literature contains accounts of the evaluation of such procedures in isolated organs and in experimental animals. Recently Bansal et al (111) have established, in the rat, the potential of thulium(III) 1,4,7,10-tetrazacyclododecane-1,4,7,10-tetrakis(methylenephosphonate) as an in vivo shift reagent for $^{23}Na^+$ in liver and other organs.

### CHELATING AGENTS AS ANTIVIRAL, ANTIMICROBIAL, ANTINEOPLASIAL and ANTIPARASITIC CHEMOTHERAPEUTICS

The extent to which inorganic biochemistry can be exploited has been amply demonstrated by utilizing the siderophore transport mechanism of cells as a drug-delivery system for therapies such as ß- lactams coupled to siderophores (112).

Two facts of note for a program of development of anti-viral and anti-tumour agents exploiting aspects of inorganic biochemistry are the reported absence of copper from virus-specific enzymes (113) and the low levels of essential trace elements in tumours in comparison to the surrounding healthy tissue (114). The recognition that inhibition of intracellular metalloenzymes by chelating agents might lead to exploitable anti-viral and cytotoxic chemotherapy has prompted a great many investigations (115). Whereas many of these studies have been restricted to laboratory evaluations, some have been used prophylactically and of particular note was the beneficial administration of thiosemicarbazones to people exposed to smallpox (116). Investigations of the anti-microbial aspects of various chelating agents were among some of the earliest uses of chelating agents, such as 8-hydroxyquinoline (HQ, oxine), outside the analytical chemistry laboratory. Although some of these early anti-microbial pharmaceutical preparations have now been withdrawn from the national pharmacopoeia of many richer countries, their uses still continue in others because they are cheap drugs. In many cases these studies have been restricted to pre-clinical investigations but in a few cases the chelating agents have been used to counter infections of humans. Foremost amongst these was the use of 5-methyl-8- hydroxyquinoline to counter cholera (117) and the use of Cl/I-HQs as amoebicides (118).

A variety of target metalloenzymes, and no doubt a variety of intracellular redoxes, make it difficult to interpret the cytotoxic action of the various chelating agents which have been screened. Investigations of the cytotoxic potential of chelating agents has continued with them formulated as metal-free preparations, as chelates of essential trace elements, particularly first row transition elements (119). Various bipyridyl chelates of several of these elements have been screened by Franchetti and his colleagues for anti-tumour and anti-fungal properties (120-121). Inactivation of ribonucleoside diphosphate reductase by chelation of Fe(II) is a frequently suggested mechanism of chelating agents such as thiosemicarbazones. The cytotoxic effects of omadine (1-hydroxypyridine-2-thione) have been attributed to the chelation of iron (122).

Among one of the earliest developments in anti-neoplasial chelation therapy was the development at the Imperial Cancer Research Fund in London of (±)-1,2-bis(3,5-dioxopiperazin-1-yl)propane (Fig. 12, ICRF 159, R=$CH_3$). Investigations of the structure activity relationships of some close congeners showed that ICRF 192 (Fig. 12, R=$C_2H_5$-) was inactive. In investigations of the sequestration of $Mg^{2+}$, $Ca^{2+}$, $Mn^{2+}$, $Fe^{3+}$, $Ni^{2+}$, $Cu^{2+}$ and $Zn^{2+}$ by the diacid diamide hydrolysis products of ICRF 159 and ICRF 192, the only significant difference which could be shown was the weaker affinity of the hydrolysis product of ICRF 159 for $Zn^{2+}$ (123).

Several studies have investigated the chemotherapeutic value of DDC for HIV. In Germany an extensive clinical study of patients receiving DDC intravenously has shown that it delays progression of HIV infection at different sites and also acts to suppress secondary pathogens (124).

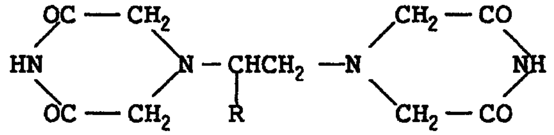

Fig. 12 Basic structure of ICRF 159 and ICRF 192

The recognition that proliferating cells have a high requirement for iron and the inhibition of a variety of malignant cell lines by DFOA led to further evaluations of ligands synthesized by Hider. These studies showed that 3-hydroxypyridin-4-ones could bring neoplastic cells to a high population of synchronised proliferating cells with enhanced sensitivity to cell cycle-specific anti-tumour agents (125).

In about the last ten years chemotherapy to counter malaria has been extended to the use of HQ derivatives (126) and ferric-selective chelating agents. In clinical studies intravenous infusion of DFOA has been shown to hasten the clearance of parasitemia (127-128) and of particular benefit was the raising from coma of children with cerebral malaria (127). Vanzyl et al (129) have proposed that the anti-malarial action is due not only iron deprivation but also to the removal of iron from the haemozoin crystal resulting in free radical generation and parasite death. Enhancement of the lipophilicity of DFOA enhances the permeation of the chelating agent through the membrane of the parasite (130). The oral uptake of 3-hydroxypyridin-4-ones could simplify anti-malarial chelation therapy (131).

### FREE RADICALS

For several years it has been recognised that oxygen radicals and other activated oxygen species, sometimes collectively identified as *reactive oxygen species* (ROS), are involved in tissue injury and the progression of several diseases (132). ROS are now implicated as the causative agents of aging, cancer, multiple sclerosis, Parkinson's disease and other diseases. An increased radical generation and lipid peroxidation is perhaps the mechanism by which many xenobiotics such as paraquat, ethanol, carbon tetrachloride and others exert toxicity. Research into these types of free radials has become so extensive that at least two research journals are devoted to the study of free radicals in medical sciences. The involvement of several of the essential trace elements, particularly in metalloproteins and as low molecular weight complexed species, in the chemistry ROS has led to the use of several chelating agents in probing the chemistry of ROS. The ROS, which include the superoxide anion ( $\cdot O_2^-$), the hydroxyl radical ( $\cdot OH$), hydrogen peroxide and singlet oxygen ( $\cdot O_2$), are generated inside cells as a result of normal electron transport or respiration. A variety of processes ranging from normal cellular function through metabolism of xenobiotics to ionizing radiation also lead to their formation.In a recent review Buettner (133) has presented a hierarchy of free radicals and anti-oxidants.

The inter-relationships of free radicals and catalytic metal ions in human diseases have been reviewed in detail (134). Investigations of metal ions and chelating agents in the chemistry of ROS have been occurring for several years. A wide variety of topics have been studied and have ranged from the formation of the hydroxyl radical in neutral solutions, from the reduction of hydrogen peroxide by chelated iron, as in the following equation:

$$Fe(II) + H_2O_2 \longrightarrow Fe(III) + OH^- + HO$$

to examination of chelated divalent transition elements as models of the enzyme superoxide dismutase (SOD).

The formation of the hydroxyl radical in more neutral solutions, in strongly acidic solutions it is the Fenton reaction, has been of interest for some years because of the recognition that tissue damage occurs on the formation of ROS in microcirculation after re-starting blood flow after cardiac arrest, stroke, organ transplant and haemorrhagic shock (135). Several publications in the proceedings of *Fifth Conference on Superoxide and Superoxide Dismutase*, published in 1991, have dwelt on a variety of aspects of chelation and oxygen radicals (136).

Among the topics included were: (i) the demonstration that deoxyribose damage was caused by converting $e^-_{aq}$ into HO· in the presence of Fe(II), Fe(III) and Cu(II); (ii) the SOD-like activity of Cu(II) complexed by carnosine, anserine and homocarnosine; (iii) determination of the SOD-like activity of Cu(II) complexed by cimetidine, an anti-peptic ulcer drug; (iv) oxidative degradation of DNA and other biological macromolecules by catecholamines in the presence of $Cu^{2+}$.

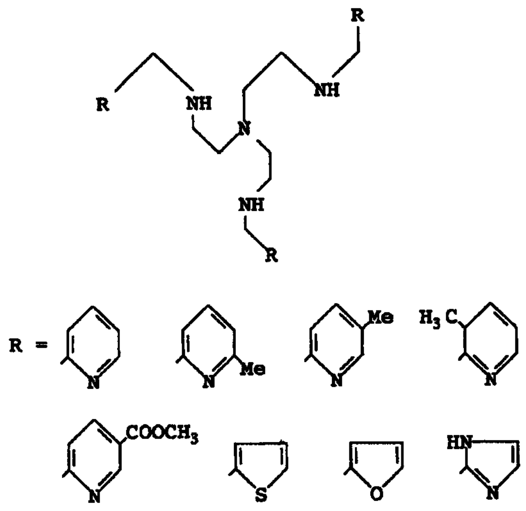

Fig 13. Substituted Tris[2-aminoethyl]amines screened for SOD activity at their Fe(III) and Cu(II) chelates.

The number of diseases in which ROS are implicated had by late 1989 exceeded sixty or more (137) and such gathering pace in investigations of ROS-triggered diseases is in turn leading to further investigations of roles for chelating agents in studying these diseases. Nagano et al (138), who have briefly summarized the various forms of complexed copper which have SOD-like activity, have synthesized a series of polyamines (Fig. 13) and shown the copper (II) chelates to have lower SOD activity than Fe(III) chelates. Several studies have demonstrated that DFOA can ameliorate tissue damage but its value is somewhat limited because of its short circulating half-life. Evaluation of DFOA- starch conjugate in ischemic rats demonstrated its prolonged circulation as well as a reduction in microvascular failure (139).

In summary, there can be little doubt that chelating agents will still play a considerable role in elucidating the inorganic biochemistry of the elements and also in combating some diseases.

**ACKNOWLEDGEMENTS**
I wish to thank my wife Maureen for supplying me with the on-line data used in Table 1.

### REFERENCES

1. H. H. Sky-Peck, *Clin. Physiol. Biochem.*, **4** (1986) 99.
2. R. L. Nelson, *Anticancer Res.*, **7** (1987) 259.
3. M. A. Atkinson & N. K. McLaren, *Sci. Amer.*, **263** (1990) 42.
4. Y. Shechter, *Diabetes*, **39** (1990) 1.
5. J. H. McNeill, V. G. Yuen, H. R. Hoveyda & C. Orvig, *J. Med. Chem.*, **35** (1992) 1489.
6. V. Sitprija, K. Tungsanga, P. Tosukhowong, N. Leelhaphunt, D. Kruerklai, P. Sriboonlue & O. Saew, *Miner. Electrolyte Metab.*, 19 (1993) 51.
7. C. Das, P. V. Kulkarni, A. Constantinescu, P. Antich, F. R. Blattner & P. W. Tucker, *Proc. Natl. Acad. Sci. USA*, **89** (1992) 9749.

8. M. G. Cherian, P. C. Huang, C. D. Klaassen, Y. P. Liu, D. G. Longfellow & M. P. Waalkes, *Cancer Res.*, **53** (1993) 922.
9. U. Lindh, *Nucl Methods Phys Res*, **B77** (1993) 261.
10. R. A. Bulman, *Structure & Bonding*, **67** (1987) 93.
11. R. C. Hider & A. D. Hall, *Progr. Med. Chem.*, **28** (1991) 41.
12. M. M. Jones, *Crit. Rev. Toxicol.*, **21** (1991) 209.
13. A. Funk & P. V. Divekar, *Can. J. Microbiol.*, **5** (1959) 317.
14. J. P. Michael & G.Pattenden, *Angew. Chemie. Inter. Ed.*, **32** (1993) 1.
15. J. H. Waite, *Anal. Biochem.*, **192** (1991) 429.
16. B. M. Eriksson & M. Wikstrom, *J. Chromatogr.*, **567** (1991) 1.
17. J. A. Fernandez-Pol, *Cell*, **14** (1978) 489.
18. D. E. Douglas, *J. Lipid Res.*, **32** (1991) 553.
19. J. R. J. Sorenson, *Free Rad. Biol. Med.*, **13** (1992) 593.
20. P. M. May, P. W. Linder & D. R. Williams, *J C S Dalton Trans*, (1977) 588.
21. F. Kieffer, In *Metals and Compounds in the Environment* (G. Merian (ed)), VCH Weinheim (1991) p. 481.
22. R. Michel, *Fresenius Z. Anal. Chem.*, **317** (1984) 451.
23. J. R. Duffield, P. M. May &. D. R. Williams, *Trace Element Metabolism In Man & Animmals, Proc. Int. Symp. 4th.*, (1981) 152.
24. S. Okuyama, S. Hashimoto, H. Aihara, W. M. Willingham & J. R. J. Sorenson, *Agents Actions*, **21** (1987) 130.
25. J. R. J. Sorenson, L. S. F. Soderberg, L. W. Chang, W. M. Willingham, M. L. Baker, J. B. Barnett, H. Salari & K. Bond, *Eur. J. Med. Chem.*, **28** (1993) 221.
26. S. Kojima & M. Jay, *Eur. J. Nucl. Med.*, **13** (1987) 366.
27. K. Palm & G. Hallmans, *Epilepsia*, 23 (1982) 453.
28. K Weismann, K, Knudsen, H. Hoyer, *J. Invest. Dermatol.*, **71** (1978) 396.
29. D. Chancel, J.-P. Oudinet, M.-P. Nivez & R. Ardaillou, *Biochem. Pharmacol.*, **31** (1982) 367.
30. S. Goldstein & G. Czapski G, *Free Rad. Res. Commun.*, 12/13 (1991) 205-210.
31. H. Kozlowski, T. Kowalik-Jankoska, A. Anouar, P. Decock, J. Spychala, J. Swiatek & Ganadu M.-L., *J. Inorg. Biochem.*, **48** (1992) 233.
32. J. Koropatnick, M. Liebbrandt & C. G. Cherian, *Radiat. Res.*, **119** (1989) 356.
33. K. H. Summer, D. Klein, N. Deruiter & J. Abel, *Biol. Trace Elem. Res.*, **21** (1989) 165.
34. X. Xiong, K. Arizano, S. H. Garrett, F. O. Brady, *FEBS Letts.*, **299**, (1992) 192.
35. N. Mutoh, M. Kawabata & Y. Hayashi, *Biochem. Biophys. Res. Commun.*, 176 (1991) 1068.
36. R. A. Bulman, In *Trace Metals and Fluoride in Bones and Teeth*, (N. D. Priest & F. L Van De Vyver (eds), CRC Press, Boca Raton (1990) pp. 271-306.
37. J. Doluiso & A. Martin, *J. Med. Chem.*, **6** (1963) 16.
38. S. Adami, G, Guarrera, F. Montesanti, S. Garavelli, S. Rosini, V. Lo Casicio, *Calcif. Tiss. Res.*, **39** (1986) 226.
39. M. C. de Vernejoul, A. Pointillart, C. Bergot, J. Bielakoff, C. Moireux, A. M. Laval Jeanert, L. Miravet,*Calcif Tiss Res*, 40 (1987) 160.
40. W. A. Volkert, B. Edwards, J. Simon, D. A. Wilson, E. H. McKenzie, P. Oberle & R. A.Holmes, *Nucl. Med. Biol.*, **13** (1986) 49. 41.P. Foley, NIOSH/HEW Press release on lead and chelating agents, March 1976.
42. FDA Memorandum on penicillamine, Department of Health Education and Welfare, May 1976.
43. *FDA Drug Bulletin*, **6** (1976) 26.
44. C. W. Jones, C. W. Mays, G. N. LLoyd & S. M. Packer, *Radiat. Res.*, 107 (1986) 296.
45. D. J. Brown, J. K. Dawborn, K. M. Ham & J. M. Xipell, *Lancet*, **ii** (1982) 342.
46. R. S. Arze, J. S. Parkinson, N. E F. Cartlidge, P. Britton & M. K. Ward, *Lancet*, **ii** (1981) 116.

47. P. Ackrill, A. J. Ralston, J. P. Day & K. C. Hodge, *Lancet*, **ii** (1980) 692.
48. D. R. C. Mclachlan, A. J. Dalton, T. P. A. Kruck, M. Y. Bell, W. L. Smith, W. Kalow & D. F. Andrews, *Lancet*, 337 (1991) 1304-1308.
49. J. P. Landsberg, B. McDonald & F. Watt, *Nature*, **360** (1992) 65.
50. T. P. A. Kruck, *Nature*, **363** (1993) 119.
51. V. Volf, R. Burgada, K. N. Raymond & P. W. Durbin, *Int. J. Radiat. Biol.*, **63** (1993) 785.
52. K. Abu-Dari, T. B. Karpishin & K. N. Raymond, *Inorg. Chem.*, **32** (1993) 3052.
53. J. Porter, *Eur. J. Haematol.*, **43** (1989) 271.
54. R. C. Hider, G. J. Kontoghiorges & J. Silver, *U K Patent GB 2 118 176, 1983; 2 136 807, 1984; 2 146 990, 1984,*
55. M. Ghisletta, H. P. Jalett, T. Gerfin, V. Gramlich & K. Hegetschweiler, *Helv. Chim. Acta*, **75** (1992) 2233.
56. T. Kradolfer & K. Hegetschweiler, *Helv. Chim. Acta*, **75** (1992) 2243.
57. R. G. Stevens & D. R. Kalkwarf, *Environ. Health Perspect.*, 87 (1990) 291-300.
58. H. Essien & K. J. Hwang, *Biochim. Biophys. Acta*, **944** (1988) 329.
59. S. W. A. Bligh, C. A. Harding, P. J. Sadler, R. A. Bulman, G. M. Bydder, J. M. Pennock, J. D. Kelly, I. A. Latham & J. A. Marriott, *Magnetic Res. Med.*, **17** (1991) 516.
60. S. W. Schwarz, C. J. Mathias, J. Y. Sun, W. G. Dilley, S. A. Wells, A. E. Martell & M. J. Welch, *Nucl. Med. Biol.*, **18** (1991) 477.
61. C. F. Meares & T. G. Wensel, *Acc. Chem. Res.*, **17** (1984) 202.
62. C. Mottahennessy, R. M. Sharkey & D. M. Goldenberg, *Appl. Radiat. Isot.*, **42** (1991) 421.
63. C. J. Mathias, Y. Sun, J. M. Cornett, G. W. Philpot, M. J. Welch, A. E. Martell, *Inorg. Chem.*, **29** (1990) 1475.
64. S. V. Deshpande, R. Subramanian, M. J. Mccall, S. J. Denardo, G. L. Denardo & C. F. Meares, *J. Nucl. Med.*, **31** (1990) 218.
65. M. K. Moi, S. J. Denardo & C. F. Meares, *Cancer Res.*, **50** (1990) S789.
66. M. Roselli, J. Schlom, O. A. Gansow, M. W. Brechbiel, S. Mirzadeh, C. G. Pippin, D. E. Milenic & D. Colcher, *Nucl. Med. Biol.*, **18** (1991) 389.
67. E. L. Kramer, S. J. Denardo, L. Liebes, L. A. Kroger, M. E. Noz, H. Mizrachi, Q. A. Salako, P. Furmanski, S. D. Glenn, G. L. Denardo & R. Ceriani, *J. Nucl. Med.*, **34** (1993) 1067.
68.Y. W. Ebright, Y. Chen, R. D. Ludescher & R. H. Ebright, *Bioconjug. Chem.*, **4** (1993) 219.
69. D. T. Reardan, C. F. Meares, D. A. Goodwin, M. McTigue, G. S. Davis, M. R. Stone, J. P. Leung, R. M. Bartholomew & J. M. Frincke, *Nature*, **316** (1985) 265.
70. M. W. Sundberg, C. F. Meares, D. A. Goodwin, & C. I. Diamanti, *J. Med. Chem.*, **17** (1974) 1304.
71. J. F. W. Keana & J. S. Mann, *J. Org. Chem.*, **55** (1990) 2868.
72. J. Altman, N. Shoef, M. Wilchek & A. Warshawsky, *J. Chem. Soc. Perkin Trans. I*, **I** (1984) 59.
73. A.Warshawsky, J. Altman, N. Kahana, R. Arad- Yellin, A. Deshe, H. Hasson, N. Shoef & H. Gottlieb, *Synthesis*, (1989) 825.
74. R. A. Bulman, J. K. Nicholson, D. P. Higham & P. J. Sadler, *J. Am. Chem. Soc.*, **106** (1984) 1118.
75. R. A. Bulman, N. Jobanputra, R. Kuroda, A. McKinnon & P. J. Sadler, *Inorg. Chem.*, **26** (1987) 2483.
76. R. A. Bulman, *Plzen Lek Sborn Suppl*, **49** (1985) 87.
77. D. J. Sawyer &. J. E. Powell, *Polyhedron*, **8**, (1989) 1425.
78. J. P. L. Cox, A. S. Craig, I. M. Helps, K. J. Jankowski, D. Parker, M. A. W. Eaton, A. T. Millican, K. Millar, N. R. A. Beeley & B. A. Boyce, *J. Chem. Soc.- Perkin Trans. I*, (1990) 2567.
79. M. S. Konings, W.C. Dow, D. B. Love, S. C. Quay, S. M. Rocklage, *Inorg. Chem.*, **29** (1990) 1488.

80. C. H. Cummins, E. W. Rutter & W. A. Fordyce, *Bioconjug. Chem.*, **2** (1991) 180.
81. L. C. Washburn, T. T. H. Sun, Y. C. C. Lee, B. L. Byrd, E. C. Holloway, J. E. Crook, J. B. Stubbs, M. G. Stabin, M. W. Brechbiel, O. A. Gansow & Z. Steplewski, *Nucl. Med. Biol.*, **18** (1991) 313.
82. D. D. Dischino, E. J. Delaney, J. E. Emswiler, G. T. Gaughan, J. S. Prasad, S. K. Srivasta, M. F. Tweedle, *Inorg. Chem.*, **30** (1991) 1265.
83. A. D. Sherry, R. D. Brown III, C. F. G. Geraldes, S. H. Koenig, K.-T Kuan & M. Spiller, *Inorg. Chem.*, **28** (1989) 620.
84. K. P. Pulukkody, T. J. Norman, D. Parker, L. Royle & C. J. Broan, *J. Chem. Soc. - Perkin Trans. II*, (1993) 605.
85. R. Y. Tsien, *Nature*, 290 (1981) 527.
86. N. R. Aiken, J. D. Satterlee & W. R. Galey, *Biochim. Biophys. Acta*, **1136** (1992) 155.
87. S. R. Adams. J. P. Y. Kao & R. Y. Tsien, *J. Am. Chem. Soc.*, **111** (1989) 7957.
88. R. Y. Tsien, T. J. Rink, *Cell Calcium*, **6** (1985) 145.
89. T. Mohri & Y. Hamaguchi, *Cell Struct. Funct.*, **16** (1991) 157.
90. B. E. Sandström, S. L. Marklund, *Int. J. Radiat. Biol.*, **62** (1992) 115.
91. H. G. Meyer-Brunot & H. Keberle, *Am. J. Physiol.*, **214** (1968) 1193.
92. P. E. Hallaway, J. W. Eaton, S. S. Panter, B. E. Hedlund, *Proc. Natl. Acad. Sci. USA*, **86** (1989) 10108.
93. M. Rivera, D. J. Levine, H. V. Aposhian & Q. Fernando, *Chem. Res. Toxicol.* **4** (1991) 107.
94. G. S. Ding & Y. Y. Liang, *J. Appl. Toxicol.*, **11** (1991) 7.
95. E. Chiotellis & A. Varvarigou, *Int. J. Nucl. Med. Biol.*, **7** (1980) 1. 96.E. Deutsch, A. R. Ketring, K. Libson, J.-L. Vanderheyden W. W. Hirth, *Nucl Med Biol*, **16** (1990) 191.
97. S. Jurisson, D. Bernint, W. Jia & D. Ma, *Chem Rev*, **93** (1993) 1137.
98. V. P. Torchilin & A. L. Klibanov, *Crit. Rev. Ther. Drug Carrier. Syst.*, **7** (1991) 275.
99. M. F. Tweedle, *J. Alloys Compounds*, **180** (1992) 317.
100. H. P. Niendorf, *Diagn Imaging*, **6** (Suppl) (1988) 16.
101. J. C. Dinger, *Contrast Media in MRI, International Workshop Berlin*, Medicom, Bussum (1990).
102. K. O. Lim, D. D. Stark, P. T. Leese, A. Pfefferbaum, S. M. Rocklage & S. C. Quay, *Radiology*,
103. O. Clement, A. Muhler, V. Vexler, Y. Berthezene & R. C. Brasch, *Invest. Radiol.*, **27** (1992) 612.
104. S. K. Kim, G. M. Pohost & G. A. Elgavish, *Bioconjug. Chem*, **3** (1992) 20.
105. A. Najafi, E. G. Amparo & F. R. F. Johnson, *J. Labelled Compd. Radiopharm.*, **24** (1987) 1131.
106. A. B. Baxter, S. Melnikoff, D. P. Stites & R. C. Brasch, *Investig. Radiol.*, **26** (1991) 1035.
107. G. Schuhmanngiampieri, H. Schmittwillich, T. Frenzel, W. R. Press & H. J. Weinmann, *Invest. Radiol.*, **26** (1991) 969.
108. S.-c. Wang, M.G. Wikström, D. L. White, J. Klaveness, E. Holtz, P. Rongved, M. E. Moseley & R. D. Brasch, *Radiology*, **175** (1990) 483.
109. O. L. M. Salonen, *J. Comput. Assist. Tomogr.*, **14** (1990) 912.
110. J. L. Sessler, T. D. Mody, G. W Hemnici, & V. Lynch, *Inorg. Chem*, **32** (1993) 3175.
111. N. Bansal, M. J. Germann, V. Seshan, G. T. Shires, C. R. Malloy & A. D. Sherry, *Biochemistry*, **32** (1993) 5638.
112. M. J. Miller & F. Malouin, *Acc. Chem. Res.*, **26** (1993) 241.
113. D. D. Perrin & H. Stünzi, *Pharm. Ther.*, **12** (1981) 255.
114. O. Warburg, *Science*, **123** (1959) 309.
115. D. D. Perrin, *Chemotherapy*, **6** (1975) 209.
116. D. Bauer, C. St. Vincent, C. Kempe & A. Dounie, *Lancet*, **ii** (1963) 494.
117. A. Zribi & M. S. B. Rachid, *Bull. Soc. Pathol. Expt.*, **66** (1973) 597.
118. W. W. Blacow, *Martindale: The Extra Pharmacopoeia*, The Pharmaceutical Press, London, (1972).

119. M. Das & S. E. Livingstone, *Br. J. Cancer*, **37** (1978) 466.
120. G. Cristalli, P. Franchetti, M. Grifanti, S. Ripa, *Il Farmaco*, **41** (1986) 499.
121. G. Cristalli, P. Franchetti, E. Nasini, S. Vittori, M. Grifanti, A. Arzi, E. Lepri, S. Ripa, *Eur. J. Med. Chem.*, **23** (1988) 301.
122. G. J. Kontoghiorghes, A. Piga, A. V. Hoffbrand, *FEBS Letts.*, 204 (1986) 208.
123. Z. X. Huang, P. M. May, K. M. Quinlan, D. R. Williams & A. M. Creighton, *Agents Actions*, **12** (1982) 536.
124. E. C. Reisinger, P. Kern, M. Ernst, P. Bock, H. D. Flad & M. Dietrich, *Lancet*, **335** (1990) 679.
125. K. P. Hoyes, R. C. Hider & J. B. Porter, *Cancer Res.*, **52** (1992) 4591. 126.L. W. Scheibel & A. Adler, *Mol. Pharm.*, **20** (1981) 218.
127. V. Gordeuk, P. Thuma, G. Brittenham, C. Mclaren, D. Parry, A. Backenstose, G. Biemba, R. Msiska, L. Holmes, E. McKinley, L. Vargas, R. Gilkeson & A. A. Poltera, *New Engl. J. Med.*, **327** (1992) 1473.
128. D. Bunnag, A. A. Poltera, C. Viravàn, S. Looareesuwan, K. T. Harinasuta & C. Schindlery, *Acta Tropica*, **52** (1992) 59.
129. R. L. Vanzyl, I. Havlik, E. Hempelmann, A. P. MacPhail & L. McNamara, *Biochem. Pharmacol.*, **45** (1993) 1431.
130. M. Loyevsky, S. D. Lytton, B. Mester, J. Libman, A. Shanzer & Z. I. Cabantchik, *J. Clin. Invest.*, **91** (1993) 218.
131. C. Hershko, V. R. Gordeuk, P. E. Thuma, E. N. Theanacho, D. T. Spira, R. C. Hider, T. E. A. Peto, G. M. Brittenham, *J. Inorg. Biochem.*, **47** (1992) 267.
132. W. A. Pryor, *Free Radicals in Biology* Academic Press, New York, 1981, Vol. 1-5.
133. G. R. Buettner, *Arch. Biochem. Biophys.*, **300** (1993) 535.
134. H. Tanaka, K. Inomata & M. Arima, *J. Nutr. Sci. Vitaminol. (Tokyo)*, **39** (1993) 177.
135. S. Rahhal & H. W. Richter, *Radiat. Phys. Chem.*, **32** (1988) 129.
136. *Free Rad. Res. Comms.*, **12/13** (1991).
137. R. A. Greenwald R A, *Free Rad. Res. Commun.*, 12/13 (1991) 531.
138. T. Nagano, T. Hirano & M. Hirobe, *Free Rad. Res. Commun.*, **12/13** (1991) 221.
139. G. T. Drugas, C. N. Paidas, A. M. Yahanda, D. Ferguson & M. G. Clemens, *Circ. Shock*, **34** (1991) 278.

**Received: September 2, 1993**

